# Shared Extracellular Matrix Remodeling and Proteomic Signature in Dupuytren’s Disease and Relapsed Clubfoot Tissue

**DOI:** 10.3390/cells15110977

**Published:** 2026-05-26

**Authors:** Tomas Novotny, Adam Eckhardt, Jarmila Knitlova, Martina Doubkova, Roman Stachon, Filip Hrdina, Tatyana Kobets, Martin Ostadal

**Affiliations:** 1 Department of Orthopaedics, University J.E. Purkyne, Masaryk Hospital, 401 13 Usti nad Labem, Czech Republic; tomas.novotny@kzcr.eu; 2 Department of Histology and Embryology, Second Faculty of Medicine, Charles University, 150 06 Prague 5, Czech Republic; 3 Laboratory of Translational Metabolism, Institute of Physiology of theCzech Academy of Sciences, Videnska 1083, 142 00 Prague 4, Czech Republic; 4 Laboratory of Biomaterials and Tissue Engineering, Institute of Physiology of the Czech Academy of Sciences, Videnska 1083, 142 00 Prague 4, Czech Republic; martina.doubkova@fgu.cas.cz; 5 Faculty of Science, Charles University, Albertov 6, 128 00 Prague 2, Czech Republic; 6 Department of Orthopaedics, Bulovka Hospital, First Faculty of Medicine, Charles University, Budinova 67/2, 180 81 Prague 8, Czech Republic; roman.stachon@gmail.com (R.S.); filiphrdina93@seznam.cz (F.H.); martinostadal@yahoo.com (M.O.); 7 Metabolomics Service Laboratory, Institute of Physiology of the Czech Academy of Sciences, Videnska 1083, 142 00 Prague 4, Czech Republic; tatyana.kobets@fgu.cas.cz; 8 Laboratory of Genomics and Bioinformatics, Institute of Molecular Genetics of the Czech Academy of Sciences, Videnska 1083, 142 00 Prague 4, Czech Republic

**Keywords:** Dupuytren’s disease, Clubfoot, relapsed Clubfoot, extracellular matrix, proteomics, fibrosis, comparative study, angiogenesis, fibroproliferative diseases

## Abstract

**Highlights:**

**What are the main findings?**

- Proteomic analysis identified differentially regulated proteins in Dupuytren’s disease and relapsed Clubfoot samples relative to their control tissues.
- 12 differentially expressed ECM-related proteins were similarly dysregulated in both Dupuytren’s disease and relapsed Clubfoot tissues
- Functional enrichment and network analyses revealed partially overlapping activation of fibrosis-associated pathways in both tissues, including structural and matricellular ECM components, ECM–receptor interaction pathways, and mechanobiological processes related to tensile stress.
- Immunohistochemical analysis further supported the presence of fibrotic ECM remodeling in Dupuytren’s disease tissue.

**What are the implications of the main findings?**

- The identification of shared disease-associated ECM-remodeling patterns suggests that clinically distinct fibroproliferative conditions may involve partially overlapping molecular processes contributing to recurrent contracture formation.
- In both diseases, progress has been made in mapping the composition of specific ECM proteins of a given type of fibrosis.
- These findings support further investigation of shared biomarkers and antifibrotic therapeutic targets across fibroproliferative musculoskeletal disorders.

**Abstract:**

Although Dupuytren’s disease (DD) and relapsed Clubfoot (RC) are clinically distinct conditions, both exhibit fibrotic tissue remodeling and contracture. This exploratory study investigated whether DD and RC share molecular features associated with fibroproliferative contracture. Pathological tissues from DD nodules and contracted tissues from RC together with their respective control tissues (*n* = 6/group), were analyzed using label-free quantitative proteomics. The analysis identified 12 significantly upregulated proteins shared between both pathological conditions relative to their controls (|log_2_FC| ≥ 1, *p* ≤ 0.05). These proteins included structural, signaling and tensile stress ECM proteins. Functional enrichment and network analyses revealed partially overlapping dysregulation of pathways associated with ECM organization and degradation, ECM–receptor interaction, matricellular signaling and mechanobiological processes. In DD samples (*n* = 10), immunohistochemistry confirmed increased expression of fibrosis-associated proteins (α-SMA, TGF-β1, TGFBI, COL III, COL VI, and COL XII) (at least *p* < 0.01). Despite these similarities, differences in individual protein abundance and collagen crosslinking were observed between tissues. The findings suggest that DD and RC may share aspects of fibrotic ECM-remodeling despite differences in age, localization, and disease origin. These findings provide initial insights into shared ECM-remodeling processes, although their interpretation should consider the relatively small sample size and biological heterogeneity of the analyzed tissues.

## 1. Introduction

Fibrotic disorders can affect most tissues in the body and are typically characterized by aberrant activation of stromal cells, particularly fibroblasts and myofibroblasts, resulting in excessive and altered extracellular matrix (ECM) production, accumulation, and function [1,2]. The extracellular matrix is not only a structural component but also a dynamic regulator of cellular behavior and mechanotransduction in connective tissues [3,4,5]. Increasing evidence suggests that fibrotic disorders, despite affecting anatomically and clinically distinct tissues, share common molecular and cellular features leading to progressive tissue stiffening, contracture formation, tissue dysfunction, or organ impairment [2,6].

Dupuytren’s disease (DD) is a chronic fibroproliferative disorder of the palmar fascia, typically affecting individuals over 50 years of age [7,8]. It is driven by myofibroblast activation and excessive ECM accumulation, resulting in the formation of fibrotic nodules and cords that cause progressive fascial thickening and flexion contractures of the digits [8,9,10]. DD is also associated with other fibrotic conditions, including Peyronie’s disease [11,12] and Ledderhose disease [13,14]. The most common treatments for DD are open partial fasciectomy, needle aponeurotomy, and Clostridium histolyticum collagenase injection [15]. However, recurrence rates remain high, with approximately one-third of cases experiencing recurrence [16,17].

Idiopathic talipes equinovarus (Clubfoot) is a complex lower limb deformity characterized by plantar flexion, adduction, inversion of the foot, an increased midfoot arch, hindfoot varus, and equinus (reviewed in [18]). A mass of contracted fibrotic tissue develops between the medial malleolus, sustentaculum tali, and navicular bone [19]. This fibrotic tissue perpetuates the deformity, leading to significant mobility limitations and a general reduction in quality of life [20]. The Ponseti method, which involves serial manipulation and corrective casting, is the most widely used treatment for Clubfoot worldwide [21,22,23]. Although this method is highly effective for primary correction, relapses occur in approximately one-third of cases, particularly between ages three and six years [24]. Similar to DD, relapsed Clubfoot (RC) is characterized by myofibroblast-driven fibroproliferation and ECM remodeling [25,26,27,28,29,30].

Table 1 presents the clinical and epidemiological characteristics of DD and idiopathic (relapsed) Clubfoot, illustrating the coexistence of shared pathological features and important biological differences that together define the rationale for this study.

This study analyzes the proteomic profiles of pathological nodular tissue from DD and the relapsed Clubfoot tissue (RCT) excised during corrective surgery. To date, no proteomic or molecular investigation has compared fibrotic processes in DD and RCT, although both conditions involve pathological alterations in connective tissue.

The aim of the study is to investigate whether DD and RCT may share partially overlapping extracellular matrix-remodeling pathways associated with fibroproliferative contracture. Previous comparative proteomic studies have demonstrated that clinically distinct musculoskeletal disorders may share partially overlapping molecular signatures and extracellular matrix-remodeling pathways, supporting the concept that common fibrosis-associated mechanisms may exist independently of anatomical localization [51,52,53]. Importantly, the present study does not aim to directly compare these anatomically and developmentally different tissues. Instead, each pathological condition is analyzed relative to its respective control tissue, allowing identification of disease-associated proteomic changes within each biological context. The cross-disease comparison is therefore based on the overlap of these disease-specific changes rather than absolute protein expression differences. Our analysis is not limited to reporting significantly dysregulated proteins; instead, we integrated protein–protein interaction networks (STRING) with pathway enrichment analyses (Enrichr, KEGG) to identify coordinated biological processes. Key fibrotic markers are difficult to detect in proteomic analyses due to their low solubility, extensive crosslinking (e.g., collagens), or low abundance (e.g., TGF-β, α-SMA). Therefore, immunohistochemistry was used as a complementary approach to validate ECM-related alterations at the tissue level. These proteins were evaluated only in DD, as they had previously been identified in relapsed Clubfoot by our group [29,46].

Given the biological heterogeneity of the analyzed tissues, the present study is designed as an exploratory, hypothesis-generating analysis. Identification of shared pathological processes may support the development of more specific mechanistic hypotheses for future investigation of fibrotic ECM remodeling and facilitate the discovery of robust biomarkers. Recent regenerative and ECM-targeted therapeutic approaches further emphasize the importance of understanding mechanobiological and extracellular matrix-remodeling processes in musculoskeletal disorders, particularly for the identification of fibrosis-associated biomarkers and potential antifibrotic therapeutic targets [54,55,56,57].

## 2. Materials and Methods

### 2.1. Patient Samples

Pathological contracted tissues of relapsed Clubfoot (RCT) were obtained during surgery from the area between the medial malleolus, navicular bone, and sustentaculum tali at the medial side of the foot. The corresponding control samples (RCT control) were obtained from normal tissue at the lateral surface of the calcaneocuboid joint of the same foot (Appendix A.1—Clubfoot), as described earlier [29].

DD samples were obtained from excised contracted palmar fascia (nodules) of DD patients undergoing surgical treatment between grades Woodruff III-IV. Control samples were obtained from the physiological necropsy palmar fascia (NPF) (Appendix A.2—DD).

In all figures, DD samples were labeled: DDN (Dupuytren’s disease nodules) × NPF (necropsy palmar fascia) and RC samples: RCT (relapsed Clubfoot tissue) × RCT control.

### 2.2. Liquid Chromatography—Mass Spectrometry (nLC-MS/MS) Analysis

Tissue samples (*n* = 6; biological replicates) (Appendix A.1, 2) were about 2 mg dry weight. All samples were washed, homogenized, treated with 4 M guanidine, delipidated, and digested with trypsin, as described in our previous study [39]. All samples were purified using StageTips (MK 360 Micro-Quartz Fiber Filters; MUNKTELL & FILTRAK, Baerenstein, Germany) and analyzed by label-free mass spectrometry quantification.

Protein extracts were analyzed by label-free quantitative proteomics using nano-liquid chromatography coupled with tandem mass spectrometry (nLC-MS/MS). Nano Reversed phase column (EASY-Spray column, 50 cm × 75 µm ID, PepMap C18, 2 µm particles, 100 Å pore size, cn ES803; Thermo Fisher Scientific, Sunnyvale, CA, USA) was used for LC/MS analysis [58]. Mobile phase buffer A was composed of water and 0.1% formic acid (Fischer Scientific, Loughborough, UK). Mobile phase B was composed of acetonitrile and 0.1% formic acid (Fischer Scientific, Loughborough, UK). Samples were loaded onto the trap column (C18 PepMap100, 5 μm particle size, 300 μm × 5 mm, cn 160454; Thermo Fisher Scientific, Sunnyvale, CA, USA) for 4 min at 18 μL/min loading buffer was composed of water, 2% acetonitrile, and 0.1% trifluoroacetic acid (Fischer Scientific, Loughborough, UK). Peptides were eluted with Mobile phase B gradient from 4% to 35% B in 60 min. Eluting peptide cations were converted to gas-phase ions by electrospray ionization and analyzed on a Thermo Orbitrap Fusion (Thermo Scientific, Waltham, MA, USA). Survey scans of peptide precursors from 350 to 1400 m/z were performed in Orbitrap at 120K resolution (at 200 m/z) with a 5 × 105 ion count target. Tandem MS was performed by isolation at 1.5 Th with the quadrupole, HCD fragmentation with normalized collision energy of 30, and rapid-scan MS analysis in the ion trap. The MS2 ion count target was set to 104, and the max injection time was 35 ms. Only those precursors with charge state 2–6 were sampled for MS2. The dynamic exclusion duration was set to 45 s with a 10 ppm tolerance around the selected precursor and its isotopes. Monoisotopic precursor selection was turned on. Each biological sample was analyzed in a single LC-MS/MS run, in accordance with standard practice for label-free quantitative discovery proteomics. The reproducibility of the Orbitrap Fusion platform for LFQ proteomics is well documented in the literature, with inter-run CVs consistently below 10% for quantified proteins. The instrument was run in top speed mode with 2 s cycles [59].

### 2.3. Proteomic Data Analysis

All data were analyzed and quantified using the MaxQuant software (version 2.0.3.0) [60]. The false discovery rate (FDR) was set to 1% for both proteins and peptides, and we specified a minimum peptide length of seven amino acids. The Andromeda search engine was used for the MS/MS spectra search against the Human database (downloaded from uniprot.org in January 2023, containing 20,606 entries). Enzyme specificity was set as C-terminal to Arg and Lys, also allowing cleavage at proline bonds and a maximum of two missed cleavages. Carbamidomethylation of cysteine was selected as a fixed modification, and N-terminal protein acetylation and methionine oxidation as variable modifications.

The MaxQuant output tables proteinGroups.txt and evidence.txt were analyzed in R (version 4.5.2) with the MSstats package (version 4.18.1) [61,62]. Data underwent median-based normalization and were summarized using Tukey’s polish procedure. Proteins identified by only one peptide were removed. Missing intensities were imputed with the MSstats method, except when an entire protein was absent across all runs [62]. Groups of two experimental models were compared in pairs: DDN versus NPF control; RCT versus RCT control. Differential expression was defined by |log_2_fold change| ≥ 1 (at least a two-fold change), *p* ≤ 0.05, and percentage of missing values after imputation ≤ 50%. Functional interpretation of significant proteins was carried out using online analysis with the STRING (https://string-db.org/) and ENRICHR (https://maayanlab.cloud/Enrichr/) databases (accessed on 12 March 2026).

### 2.4. Histopathological Investigation

Tissue samples (*n* = 10 per group; DDN vs. NPF) (Appendix A.2) were fixed and embedded in paraffin. Primary antibodies against alpha smooth muscle actin (anti-α-SMA, A5228, (Sigma, St. Louis, MO, USA); 1:400, 4 °C overnight), Transforming Growth Factor Beta 1 (anti-TGF-β1, ab66043 (Abcam, Cambridge, UK); 1:200, 4 °C overnight), Transforming Growth Factor Beta Induced Protein (anti-TGFBI, ab170874 (Abcam, Cambridge, UK); 1:250, 1 h RT), collagen III (anti-Col III, Sigma C7805 (St. Louis, MO, USA), 1:500, 1 h RT), collagen VI (anti-Col VI (Abcam, Cambridge, UK), ab6588, 1:200, 30 min RT), collagen XII (anti-Col-XII, SAB4500395 (Sigma, St. Louis, MO, USA); 1:50, 1 h RT) were used for detection by immunohistochemistry (IHC). Antigen retrieval, hydrogen peroxide block, protein block, secondary antibody reaction, and visualization were performed according to the Abcam protocol by applying the EXPOSE Mouse and Rabbit Specific HRP/DAB IHC Detection Kit (ab236466, Abcam, Cambridge, UK). The slides were counterstained with hematoxylin. The thresholding procedure followed a standardized methodology that our team has repeatedly used in previous histopathological and immunohistochemical studies [29,43,45,46]. The positivity of the detected antibodies was evaluated by light microscopy, and quantification was performed using image-analyzer signal thresholding (NIS-Elements AR software for non-clinical diagnostic use, version 6.20.02, Laboratory Imaging, Prague, Czech Republic). A positive control (a tissue selected according to the antibody producer’s recommendations) was used to manually define the threshold for the positive signal. The number of pixels within the signal range was then quantified from 10 independent areas of each sample, and the percentage of the positive area was calculated and compared across the experimental groups. All image analyses were performed independently by the same experienced observer, who has applied this methodology for many years. The threshold was consistently defined according to the strongest specifically immunohistochemically positive structures identified in the positive control sample slide. This thresholding approach was then applied in a standardized manner to ten distinct representative areas of each specimen. The use of a positive-control-based reference threshold and repeated measurements across multiple fields was intended to minimize subjective variability and ensure methodological consistency across all analyzed samples. For each experimental group, the same primary and secondary antibodies were used across all tissues, the same reagents were used at the same concentrations, and all incubation and development times were identical. For the purposes of the image analysis, no deconvolution was used. Both object classes (negative vs. positive) were segmented using a threshold in the RGB color space. The IHC-positive area data from both the DDN and NPF control groups were examined for normality (Shapiro–Wilk test, Q-Q plot), and an appropriate parametric or non-parametric statistical test was used to compare the data (unpaired *t*-test with Welch’s correction or Mann–Whitney test). The significance level for rejection of the null hypothesis was set at 0.05. GraphPad Prism v. 10.4.1 (GraphPad Software, Boston, MA, USA) was used for statistical analysis and graph creation.

### 2.5. High-Performance Liquid Chromatography (HPLC)

HPLC analysis of collagen crosslinks was performed as follows: All samples (RCT: *n* = 12 per group; DDN: *n* = 19, NPF: *n* = 10) (Table A2) were lyophilized (18 h) and then hydrolyzed (6N HCl, 105 °C, 18 h). The HPLC analysis was performed at HPLC Agilent 1100 (Agilent Technologies, Santa Clara, CA, USA) [63] using the protocol from Chromsystems (c.n. 48000): “Instruction manual for the HPLC analysis of Crosslinks in urine” (Chromsystems Instruments, Grafelfing, Germany) with sampling frequency 2.5 Hz. The intra-assay coefficient of variation was 5.8% and inter-assay coefficient of variation was 12.4%. The concentration of trivalent collagen crosslinks (pyridinoline) obtained by HPLC was measured in nmol/mg (mg—the dry weight of the sample). The data from both groups (RCT and RCT control; DDN and NPF) were examined for normality (Shapiro–Wilk test). A paired *t*-test was used to compare the RCT and RCT control groups, as the pathological and control tissue samples originated from different areas of the same foot. A Mann–Whitney test was used to compare the DDN and NPF groups, as the data were not normally distributed and the compared tissue samples originated from different patients. The significance level for rejection of the null hypothesis was set at 0.05. GraphPad Prism v. 10.4.1 (GraphPad Software, Boston, MA, USA) was used to perform the statistical analysis and to create graphs.

No correction for age was performed across the compared groups. Applying covariate-based correction (based on patient age) under such conditions would risk overfitting and could introduce greater analytical uncertainty than it resolves. Moreover, no sufficiently large reference proteomics dataset of healthy palmar fascia across a comparable age range currently exists in the literature, which precluded the use of external age-correction models. The potential influence of age-related molecular changes—including collagen crosslink accumulation and altered extracellular matrix remodeling—on the observed proteomic differences is acknowledged as a limitation of this study (more information about the limitations of the study is provided in Section 4 (Discussion)).

## 3. Results

### 3.1. Proteomics

**Figure 1:**
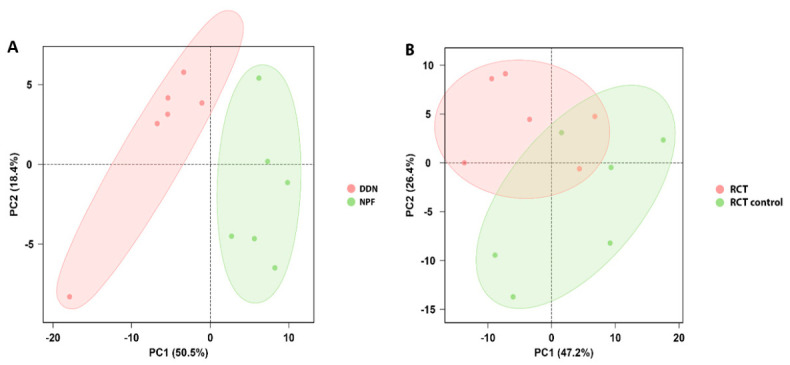
PCA of (**A**) DD comparison (DDN × NPF) and (**B**) RC comparison (RCT × RCT control). The PCA tool R package was used to perform principal component analysis and generate biplots; the default confidence interval was set to 95%.

Proteomic profiling showed high individual variability among the tested samples within experimental groups. Principal component analysis (PCA) demonstrated group separation, reflecting proteomic remodeling in DDN versus control NPF (50% of variance explained by principal component 1 (PC1)); (Figure 1). In RCT versus RCT control, PC1 illustrated extensive variance within both groups, while PC2 characterized the difference between groups (26% of explained variance).

**Figure 2:**
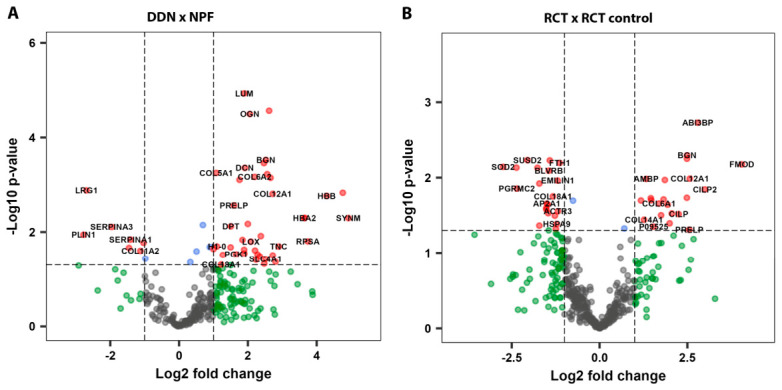
Results of differential protein expression analysis. (**A**) Comparison between DDN (the right side of the picture) and NPF (the left side of the picture); (**B**) comparison of RCT (the right side of the picture) versus the RCT control (the left side of the picture). Volcano plots show all detected proteins, regardless of significance. Red dots indicate proteins, significant by *p*-value and log_2_FC (see Methods (Section 2)); green dots depict proteins exceeding the log_2_FC threshold but not reaching the *p*-value significance threshold; gray dots show the remaining proteins.

Proteomic comparisons of tissues from both diseases revealed significantly altered concentrations of 74 proteins. Statistical comparison of protein expressions in pairs of groups revealed a narrow set of proteins with differential expression patterns: 6 downregulated and 37 upregulated proteins in DDN samples; 10 downregulated and 21 upregulated proteins in RCT (Figure 2; Supplemental Tables S1 and S2). Most of the upregulated proteins found in the RCT (12 proteins) overlapped with those upregulated in DDN (collagen type VI (alpha 1, 2, 3), type XII (alpha 1), TGF-β-induced protein, biglycan, tenascin C, fibromodulin, and asporin) (Supplemental Tables S1 and S2).

In addition to these proteins, we identified significantly higher concentrations of collagen types IV (alpha 1) and V (alpha 1), vimentin, and lysyl oxidase (LOX) in DDN versus NPF control (Supplemental Table S1) and cartilage oligomeric matrix protein, elastin microfibril interfacer 3, and versican in RCT versus RCT control (Supplemental Table S2).

### 3.2. STRING

All proteins that were found to be significant for log2 fold change (log_2_FC) ≥ 1 were analyzed for protein–protein interaction (PPI) by a Search Tool for the Retrieval of Interacting Genes and Proteins (STRING, STRING: functional protein association networks (string-db.org)).

The identified clusters were annotated according to the most significantly enriched terms. To improve readability, multiple GO (gene ontology) term labels were shortened; thus, the corresponding GO identifiers are also provided.

**Figure 3:**
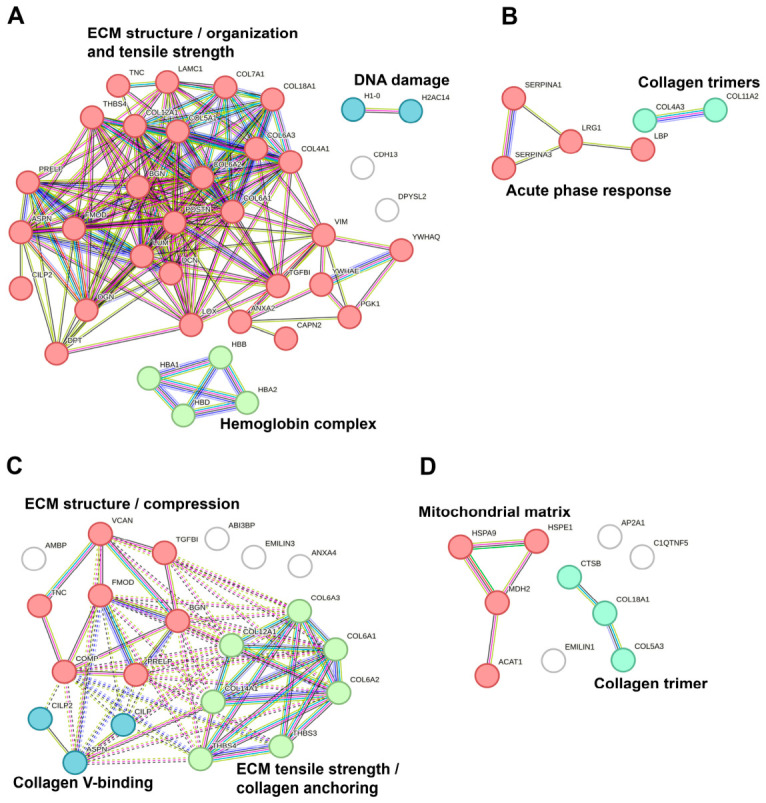
STRING protein–protein interactions. STRING protein–protein interactions between proteins upregulated in DD (**A**), NPF (**B**), RCT (**C**), and RCT control (**D**). STRING network analyses of proteins with *p* ≤ 0.05 and FC ≥ 1 are presented. Intracluster interactions are shown in solid lines, and interactions between different clusters are illustrated with dotted lines.

The STRING analysis of 37 proteins upregulated in DDN samples revealed three main clusters (Figure 3A). The first cluster comprised 29 proteins and showed significant enrichment for GO terms “ECM structure/organization/tensile strength” (GO:0005201, *p* = 1.20 × 10^−23^; GO:0030198, *p* = 5.28 × 10^−11^; GO:0030020, *p* = 7.28 × 10^−11^). The second cluster (4 proteins) was significantly enriched for “Hemoglobin complex” GO term (GO:0005833, *p* = 4.45 × 10^−10^). The third cluster contains only 2 proteins, histone subunits, and was not assigned to any GO term but the HSA (homo sapiens) “DNA Damage” term (HSA-2559586, *p* = 0.0222).

The STRING analysis of 6 proteins upregulated in NPF control identified two clusters (Figure 3B). The first cluster (4 proteins) showed significant enrichment for GO:0006953 “Acute phase response” (*p* = 0.0007). The second cluster (2 proteins) was enriched for GO:0098644 “Collagen trimers” (*p* = 0.0029).

The STRING analysis of 21 proteins upregulated in RCT revealed three main clusters (Figure 3C). The first cluster (7 proteins) showed significant enrichment for “ECM structure/Compression” (GO:0005201, *p* = 3.47 × 10^−9^ and GO:0030021, *p* = 5.51× 10^−5^). The second cluster (7 proteins) was enriched for “ECM tensile strength/collagen anchoring (GO:0030020, *p* = 9.91 × 10^−10^; GO:0030934, *p* = 0.00028). Three proteins in the third cluster were enriched for “Collagen V binding” (CL:19819, *p* = 0.0055).

The STRING analysis of 10 proteins upregulated in RCT control revealed two clusters (Figure 3D). The first cluster (4 proteins) showed significant enrichment for GO:0005759 “mitochondrial matrix” (*p* = 0.00083). The second cluster was enriched for GO:0005581 “Collagen trimer” (*p* = 0.0368).

**Figure 4:**
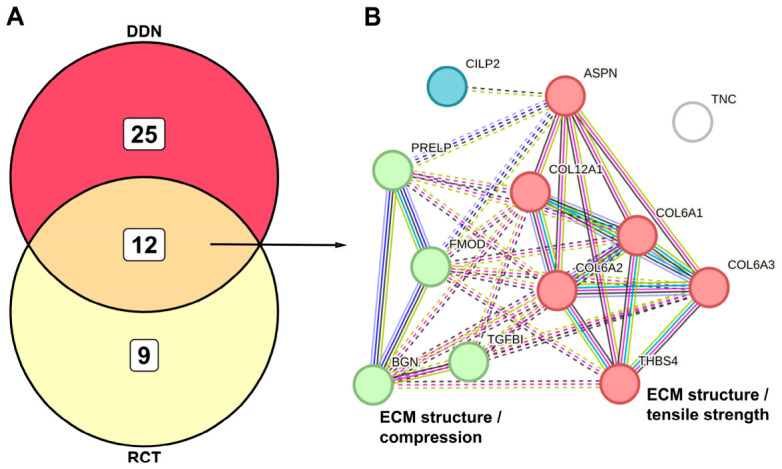
Venn diagram and STRING analysis. Venn diagram (**A**) shows the number of unique and shared proteins upregulated in DDN and RCT comparisons (vs. their controls). The overlap of 12 proteins upregulated in both comparisons is visualized by STRING protein–protein interaction analysis (**B**). Intracluster interactions are shown in solid lines, intercluster interactions are shown with dotted lines.

A subset of 12 proteins was consistently upregulated in both fibrotic DDN and RCT samples, representing a shared extracellular matrix-remodeling signature (Figure 4A). This common profile included structural collagen type VI and collagen type XII, together with several proteoglycans and small leucine-rich matrix regulators, including asporin (ASPN), biglycan (BGN), fibromodulin (FMOD), and prolargin (PRELP). The presence of cartilage intermediate layer protein 2 (CILP2) further supports altered matrix composition in fibrotic tissue. Importantly, several matricellular proteins associated with profibrotic signaling were also increased, including TGF-β–induced protein (TGFBI), thrombospondin-4 (THBS4), and tenascin-C (TNC). The STRING analysis revealed significant clustering associated with ECM structure and mechanical properties, particularly “ECM structure/tensile strength (GO:0030020, *p* = 4.23 × 10^−7^) and “ECM structure/compression” (GO:0005201, *p* = 0.006 and GO:0030021, *p* = 0.0104) (Figure 4B). Collectively, these consistently upregulated proteins indicate a fibrotic profile characterized by enhanced deposition of structural ECM components, increased matricellular signaling and alterations in the mechanical properties of the ECM.

### 3.3. ENRICHR

**Figure 5:**
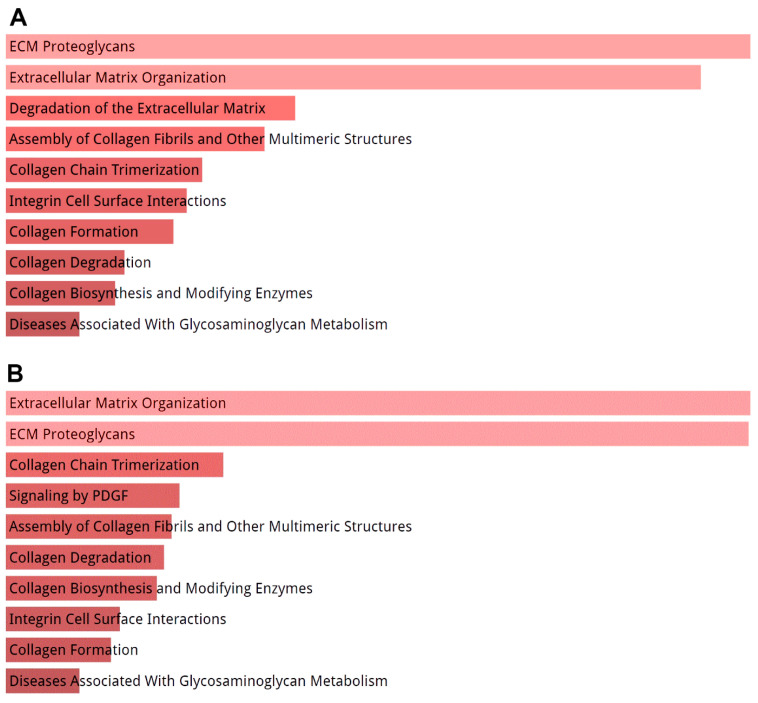
The Gene Ontology Enrichment analysis “Reactome Pathways 2024” of the set of proteins upregulated in DDN (**A**) and in RCT (**B**) revealed these 10 significantly increased Reactome pathways. The length of the horizontal bar and its color corresponds to the degree of significance in each category. All mentioned pathways have a significance *p* (FDR) less than *p* = 1.0 × 10^−6^ (which is for the last mentioned “Diseases Associated With Glycosaminoglycan Metabolism”, which is with the lowest significance FDR in both pictures), and specifically, two main pathways in both comparisons (“ECM Proteoglycans” and “Extracellular Matrix Organization” which are with the highest significance in both pictures) have significance *p* (FDR) less than *p* = 1.0 × 10^−15^.

The comprehensive gene set enrichment analysis tool Enrichr (https://maayanlab.cloud/Enrichr/enrich#; accessed on 12 March 2026). was used to provide synthesized information about protein (gene) sets. Relevant properties of two protein sets were identified (37 significantly upregulated in DDN vs. NPF control, and 21 significantly upregulated in RCT vs. RCT control (their genes)) in the specific category “Reactome Pathways 2024” (Figure 5). All significantly changed proteins were calculated (separately) in these analyses.

### 3.4. KEGG

**Figure 6:**
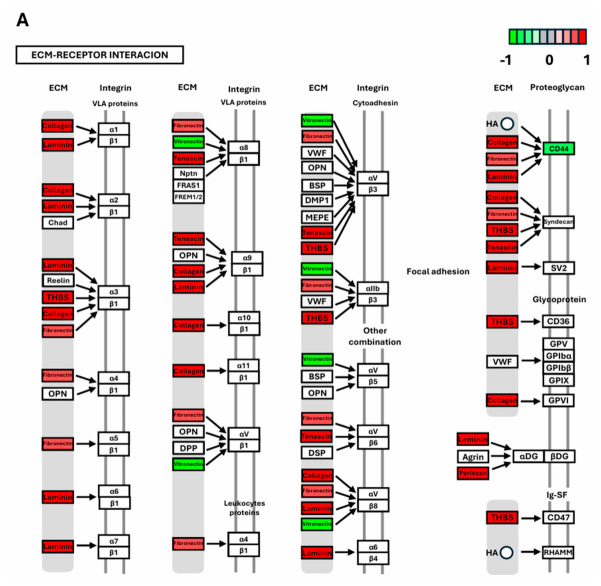

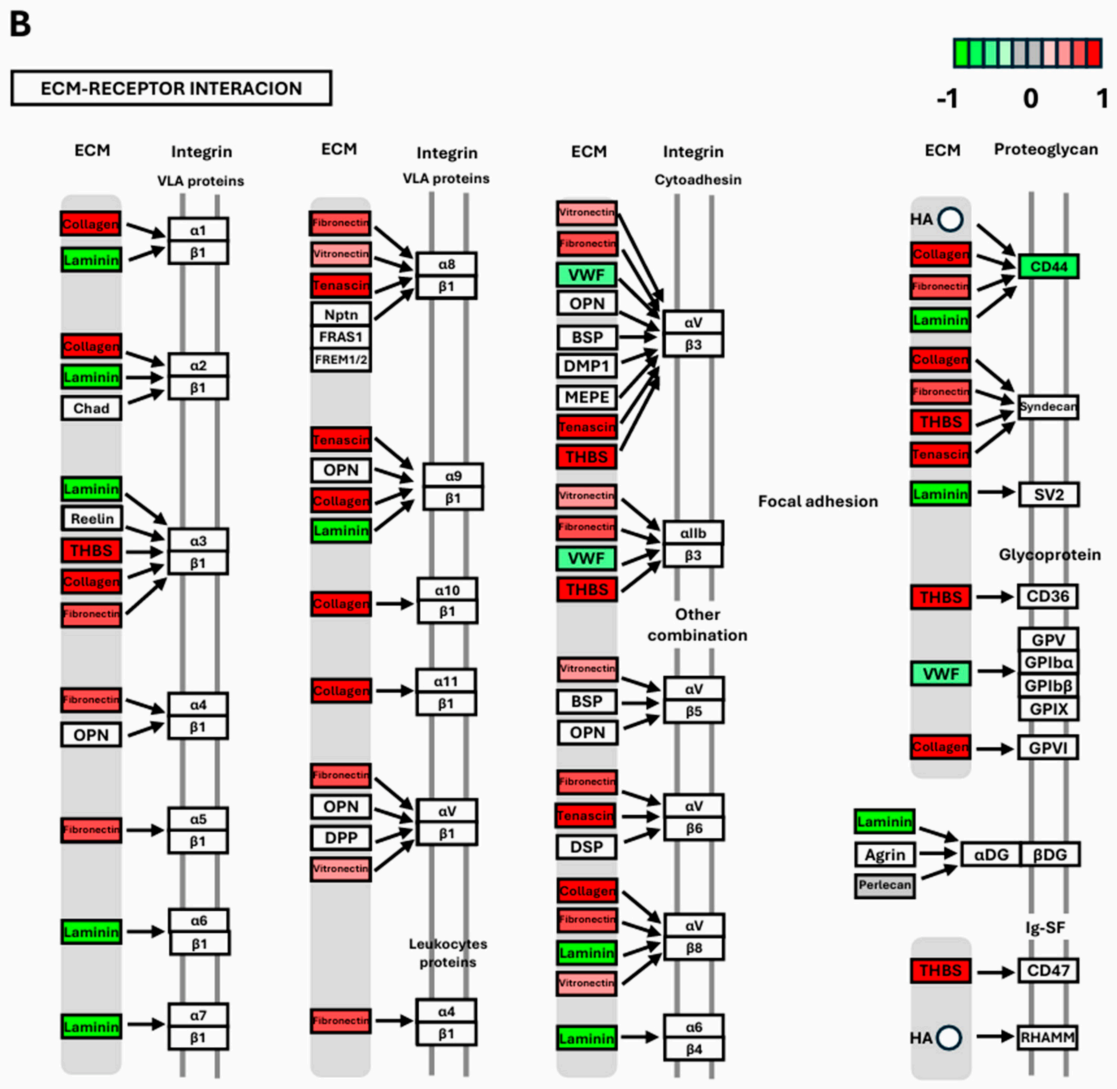
Graphic representation of changes in expression of proteins annotated to the ECM–receptor interaction KEGG pathway (hsa04512). Comparison of samples: (**A**) DDN versus NPF; (**B**) RCT versus RCT control. Color scale shows values of log_2_ fold change (log_2_FC) between groups after protein differential expression analysis in the range −1 < 0 < 1. Terminal red color indicates log_2_FC ≥ 1 (increase); terminal green color indicates log_2_FC ≤ −1 (decrease). Basic KEGG graphs were downloaded from https://www.genome.jp/ (accessed on 12 March 2026). Protein names (symbols) are shown according to the KEGG nomenclature.

The two diseases were also compared in the expression of proteins annotated to the Kyoto Encyclopedia of Genes and Genomes (KEGG) ECM–receptor interaction pathway (hsa04512) (Figure 6). Within the proteins involved in ECM–receptor interactions, we found the following proteins to be upregulated in both DD and RCT: collagen, thrombospondin (THBS), fibronectin, and tenascin. In DDN, laminin was also upregulated, whereas vitronectin was downregulated (Figure 6). In RCT, the situation was the opposite (i.e., upregulated vitronectin and downregulated laminin).

### 3.5. Immunohistochemistry (IHC)

**Figure 7:**
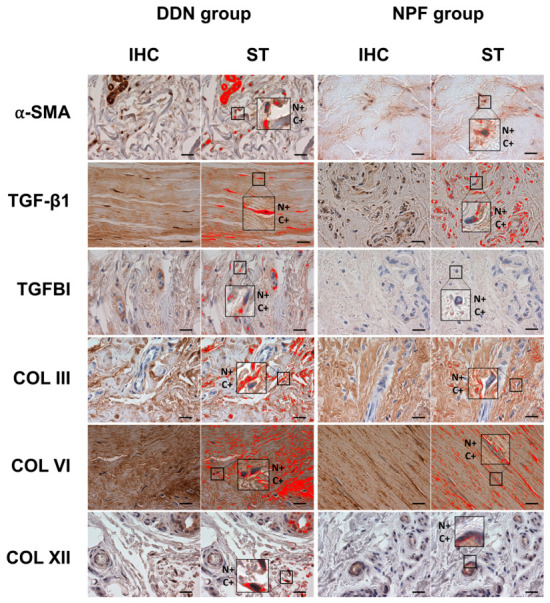
Representative images of Dupuytren’s disease immunohistochemical staining (IHC) and signal detection following thresholding (ST). The red areas in the ST columns represent the pixels analyzed after the thresholds were set. NIS element software (version 6.20.02) was used to quantify the percentage of positively stained areas relative to the total area. Alpha smooth muscle actin (α-SMA), Transforming Growth Factor Beta 1 (TGF-β1), Transforming Growth Factor Beta Induced Protein (TGFBI), collagen type III (Col III), collagen type VI (Col VI), and collagen type XII (Col XII); ST Signal Threshold, N+ Nuclear positivity, C+ Cytosolic positivity. Scale bar = 20 μm.

**Figure 8:**
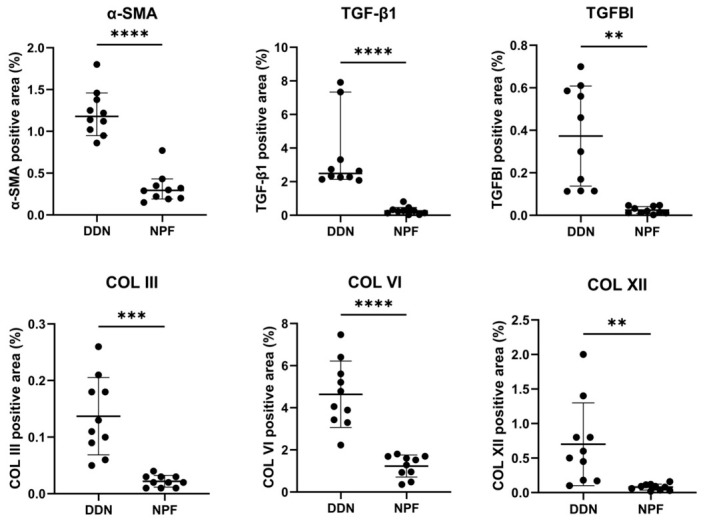
Quantitative analysis of immunohistochemical staining of DDN and NPF. The percentage of alpha smooth muscle actin (α-SMA), Transforming Growth Factor Beta 1 (TGF-β1), Transforming Growth Factor Beta Induced Protein (TGFBI), collagen type III (COL III), collagen type VI (COL VI), and collagen type XII (COL XII) positive area after immunohistochemical detection. Data (*n* = 10 per group; biological replicates) are presented as medians with 95% CI (α-SMA, TGF-β1) or means with SD (TGFBI, COL III, COL VI, COL XII). Mann–Whitney test (α-SMA, TGF-β1: **** *p* < 0.0001) or unpaired *t*-test with Welch’s correction (TGFBI: ** *p* < 0.01; COL III: *** *p* = 0.0004; COL VI: **** *p* < 0.0001; COL XII: ** *p* = 0.0096).

DDN samples showed a significant increase in α-SMA, TGF-β, TGF-β-induced protein, collagen type III, collagen type VI, and collagen type XII positive areas compared to NPF controls (Figure 7 and Figure 8).

### 3.6. Collagen Crosslinks

**Figure 9:**
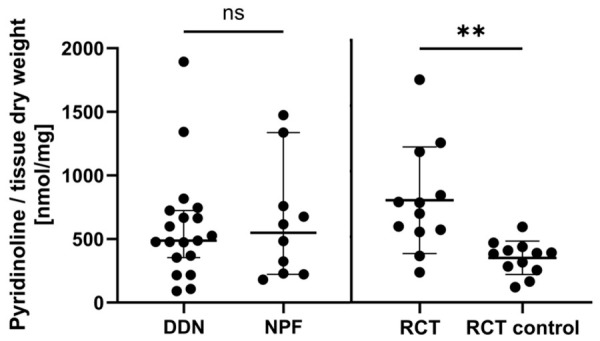
The concentration of trivalent collagen crosslinks. The concentration of trivalent collagen crosslinks (pyridinoline) in tissue samples DDN, RCT, and their controls. Data are reported as medians with 95% CI (DDN vs. NPF group) or means with SD (RCT vs. RCT control group). A Mann–Whitney test was used to compare DDN (*n* = 19; biological replicates) and NPF (*n* = 10; biological replicates) groups (ns = non-significant). A paired *t*-test was used to compare RCT (*n* = 12; biological replicates) and RCT control (*n* = 12; biological replicates) groups (** *p* = 0.0071).

Collagen crosslinking was assessed, as it reflects the degree of ECM maturation and directly contributes to increased tissue stiffness and mechanical resistance, key features of advanced fibrotic remodeling and contracture formation. A significantly higher concentration of collagen crosslinks (pyridinoline) was detected in the RCT samples in comparison with the RCT controls by the HPLC method. No significance was observed in the comparison of DDN with NPF control (Figure 9).

## 4. Discussion

Both DD and RC are characterized by the development of stiff, fibrotic, contractile tissue; however, to our knowledge, these two pathologies have not been previously compared at the proteomic level. While DD is an acquired disease of adults, considerably more prevalent and therefore more extensively studied, RC represents a unique form of fibrosis arising from a developmental disorder. The combination of proteomic profiling, immunohistochemical validation, and collagen crosslink analysis was intended to provide complementary insight into ECM-remodeling at molecular, histological, and biochemical levels. While proteomics enabled broad characterization of dysregulated proteins and pathways, immunohistochemistry supported the presence of selected fibrosis-associated proteins that were difficult to analyze by mass spectrometry. Detection of key fibrotic markers in proteomic analyses is hampered by their poor solubility, extensive crosslinking—as seen in collagens—or low abundance, as is the case for TGF-β and α-SMA. HPLC analysis of collagen crosslinks provided additional information regarding collagen maturation. The study revealed partially overlapping ECM-remodeling and fibrosis-associated pathways in both conditions.

In both diseases, most of the upregulated proteins were related to structural collagens (notably types VI and XII), small leucine-rich proteoglycans (SLRP, e.g., fibromodulin, biglycan, asporin), and matricellular proteins (e.g., TGFBI, thrombospondin 4, periostin, tenascin) (Figure 4B). STRING network analyses and Functional enrichment (ENRICHR; Reactome/GO) consistently indicated enrichment of ECM organization, collagen biosynthesis and trimerization, ECM proteoglycans, and ECM–receptor interaction pathways (Figure 3 and Figure 5). Several studies suggest that fibrosis affecting different tissues may have organ-specific triggers, while the progression of fibrosis shares core molecular pathways (reviewed in [2,64]). Proteins associated with collagen organization, matricellular signaling, and tissue stiffening are commonly involved in fibrotic remodeling across multiple tissues and diseases [2,64]. Identification of these pathways in DD and RC may be relevant for the discovery of fibrosis-associated biomarkers and may also have potential therapeutic implications.

Our findings in DD nodules are broadly consistent with previously published molecular studies demonstrating active ECM remodeling in DD nodules. A study comparing nodules and cords [65] showed that nodules represent the active site of the disease. Nodules contained a higher proportion of proliferating cells, CD68+ macrophages, and α-SMA-positive myofibroblasts. Transcriptomic analysis by Rehman et al. demonstrated significantly increased expression of collagen types I, V, and VI, as well as matrix-remodeling-associated genes (e.g., matrix metalloproteinases, their inhibitors, ADAMTS family members, tenascin C, and periostin) in DD nodules compared with palmar fascia [66,67,68]. Proteomic profiling of affected (without discrimination nodules × cords) palmar fascia revealed upregulation of collagen type 6, TGFBI, together with increased concentration of oxidative stress proteins, cytoskeletal changes, and involvement of autocrine signaling pathways [69]. More recently, studies with decellularized DD ECM demonstrated that altered matrix composition can actively promote macrophage activation and sustain myofibroblast differentiation [28]. Secretome analysis of DD-derived fibroblasts grown on decellularized DD nodules (3D DD model) detected, in addition to fibrous proteins, abundant TGF-β1, IL-6, MMPs, and collagen crosslinking enzymes [27]. Recent studies further emphasize the importance of fibroblast-derived paracrine signaling in connective tissue remodeling and repair processes [70].

Importantly, our proteomic analysis of relapsed Clubfoot tissue samples represents a unique study, as these samples are extremely rare. Previous studies on RCT have consistently reported the presence of α-SMA-positive myofibroblasts [46], increased expression of structural ECM proteins [29,37,42,43], growth factors and signaling molecules [37,45,71], and collagen crosslinking enzymes [43,46], supporting the presence of active fibroproliferative remodeling in affected tissues. The current proteomic findings are broadly consistent with these observations and further suggest that recurrent Clubfoot contracture may involve features of ongoing ECM remodeling, mechanobiological signaling, and matrix maturation processes similar to those observed in other fibrotic connective tissue disorders [2,64].

At the same time, we identified disease-specific proteins, which we believe primarily reflect the sub-stages of fibrosis and age-specific differences rather than indicating distinct fibrotic pathways. DD samples showed increased lysyl oxidase (LOX), additional collagen subtypes, osteoglycin, and decorin, suggesting active matrix maturation and stabilization, indicating a slightly more advanced stage of fibrosis. Interestingly, differential abundance of laminin (upregulated in DDN) and vitronectin (upregulated in RCT), both of which interact with integrin receptors, was detected in the KEGG analysis. Increased vitronectin levels in RCT may suggest enhanced angiogenic activity, as vitronectin is known to promote endothelial cell adhesion and migration [72,73]. Previously reported stiffness measurements in RCT [74] and DD nodule slices [27] are consistent with the concept that ECM remodeling is associated with increased tissue stiffness. However, in the present study, increased pyridinoline crosslinking was detected only in RCT compared with its control, whereas no significant difference was observed between DD nodules and NPF. Van Beuge et al. demonstrated that (pyridinoline and deoxypyridinoline) crosslink density is increased in cords compared to nodules, indicating compartment-specific maturation stages within DD [65]. This distinction is important for interpreting our collagen crosslink measurements. Several factors may explain this finding. First, nodules may represent a less mature stage of fibrosis compared with cords. Second, older NPF controls may reduce contrast, as age-related accumulation of collagen crosslinks in control tissue could mask disease-related differences. Thus, the absence of a difference in crosslink concentration does not necessarily contradict the established concept of progressive matrix stiffening in DD. We acknowledge that the higher age of the control donors may represent a potential confounding factor influencing both crosslink and proteomic comparisons. However, the availability of younger healthy palmar fascia controls is extremely limited.

In DD, the enrichment of hemoglobin-related GO term (Figure 5A) may reflect increased vascularization reported in DD [44]; however, it may also indicate minor blood contamination, as complete removal of blood from the rigid, fibrotic fascia may have been insufficient.

Chronic inflammation has long been implicated in DD [75], and inflammatory mechanisms play a crucial role in fibroproliferative disorders (reviewed in [76]). Recent work demonstrates macrophage-driven reinforcement of myofibroblast activation in response to altered ECM in DD [28]. There is very little information available about the possible presence of inflammation in RC. Our results from this study showed, among others, significantly increased levels of alpha-1-microglobulin (AMBP), Complement C1r subcomponent (P09525), and ABI family member 3 binding protein (ABI3BP) (Figure 3C). These proteins are associated with oxidative stress responses, complement activation, and the regulation of fibroblasts [77]. The presence of AMBP and complement component C1r may suggest immune-related activity; however, this finding alone does not provide direct evidence of inflammatory cell infiltration.

Several limitations should be considered when interpreting the study’s proteomic findings. First, the relatively small sample size (six biological replicates per group) limits statistical power. The availability of RCT was inherently restricted due to the rarity of surgical intervention in these patients. In addition, to ensure analysis of actively fibroproliferative DD tissue, only nodular tissue samples were included, while late-stage cord-dominant samples were excluded. As surgical treatment is most commonly performed in advanced disease stages characterized predominantly by fibrotic cords, this selection strategy further reduced the number of suitable DD specimens.

Another important limitation is the use of different control tissue types between the analyzed conditions. Although patient-matched control tissue was used for relapsed Clubfoot, these samples may not represent fully healthy tissue, as a noncontracted tissue from the same patient foot might be still biomechanically affected. Nevertheless, the use of truly healthy pediatric connective tissue controls is ethically and practically impossible. Control tissue for DD was obtained from necropsy samples due to the limited availability of healthy palmar fascia. This difference may represent a potential source of variability related to postmortem tissue changes and tissue processing conditions. Previous studies on DD have used, as a control, macroscopically unaffected fascia from the same DD patient [68,69] or tissue biopsy excised during surgery for carpal tunnel [28]. However, such tissues may still be biomechanically or molecularly affected by the disease process and therefore may not represent a truly healthy control. Despite these limitations, partially overlapping ECM-associated molecular findings were independently identified in both disease-control comparisons, supporting the biological relevance of the observed fibrosis-associated pathways. The study was focused on comparing fibrosis-associated molecular remodeling processes rather than directly comparing the two diseases themselves. Nevertheless, differences in age, anatomical localization, and tissue biology may represent potential confounding factors and should be considered when interpreting the observed overlap in extracellular matrix-related pathways.

The findings presented here, including dysregulation of extracellular matrix composition and signaling, immunohistochemical detection of several profibrotic proteins in DDN, and enhanced collagen crosslinking in RCT remain exploratory, the identified pathways may be biologically relevant in the broader context of connective tissue fibrosis and mechanobiological tissue stiffening. Similar ECM-associated remodeling processes have been described in other musculoskeletal connective tissue disorders, including tendinopathy and diabetic tendon pathology, where altered collagen organization, matrix stiffness, and chronic remodeling contribute to tissue dysfunction [78]. Identification of recurrently dysregulated ECM proteins and fibrosis-associated pathways may therefore support future investigation of candidate biomarkers and potential antifibrotic therapeutic strategies. In terms of experimental treatments for DD, the TNF-α inhibitor adalimumab has shown promising results in phase 2b clinical trials for early-stage DD [41]. Recently, experimental in vitro studies [27,40,43] have been conducted with the antifibrotic drug minoxidil. In both cases, minoxidil decreased collagen type I accumulation in DD and RC fibroblast cultures.

## Figures and Tables

**Table 1:** Clinical, epidemiological, and pathological comparison of Dupuytren’s disease and idiopathic Clubfoot. Findings marked (RC) were studied only in relapsed Clubfoot.

Feature	Dupuytren’s Disease	Clubfoot (and RC)
Affected tissue (localization)	Hand: Palmar fascia [7,8]	Leg: Soft tissues between the medial malleolus, sustentaculum tali, and navicular bone [18,31]
Typical detection age	Most commonly after age 50 [7,8]	Prenatally or at birth [18,32]
Global prevalence	~5–15% [7,33]	~0.1–0.2% [25,34]
Sex distribution (women: men)	~1:4 [35]	~1:2 [36]
Ethnic variability in incidence	Yes [7]	Yes [18]
Etiology	Unknown [8,26]	Unknown [18,23]
Main pathological cell type	Myofibroblasts [9,20]	Myofibroblasts [32]
Pro-fibrotic markers	Yes [12,27,28]	Yes (RC) [29,37,38,39,40]
Experiments with antifibrotics	adalimumab [41] minoxidil [27]	β-aminopropionitrile (RC) [42] minoxidil (RC) [40,43]
Increased angiogenesis	Yes [44]	Yes (RC) [45]
Hypoxia-related changes	Yes [44]	Yes (RC) [46]
Common treatment	Surgery or minimally invasive release [8,30,47]	Serial casting (Ponseti method) [18,22,23,48,49]
Relapse rate	~30% [16,17]	~30% [24,50]

## Data Availability

The datasets generated and/or analyzed during the current study will be available at https://zenodo.org/ (accessed on 12 March 2026) and are also available from the corresponding author.

## Supplementary Materials

The following supporting information can be downloaded at: https://www.mdpi.com/article/10.3390/cells15110977/s1. Table S1: The list of proteins with significantly changed concentration DD vs. NPF; Table S2: The list of proteins with significantly changed concentration RCT vs. RCT control.

## Abbreviations

The following abbreviations are used in this manuscript:

ASPN	Asporin
BGN	Biglycan
CILP2	Cartilage Intermediate Layer Protein 2
Col III	Collagen type III
Col VI	Collagen type VI
Col XII	Collagen type XII
DD	Dupuytren’s disease
DDN	Dupuytren’s disease nodules
ECM	Extracellular matrix
FDR	False Discovery Rate
FMOD	Fibromodulin
GO	Gene Ontology
HCD	Higher-energy Collisional Dissociation
HPLC	High-Performance Liquid Chromatography
IHC	Immunohistochemistry
KEGG	Kyoto Encyclopedia of Genes and Genomes
LC/MS	Liquid Chromatography–Mass Spectrometry
log_2_FC	Log2 Fold Change
LOX	Lysyl Oxidase
MS	Mass Spectrometry
MS2	Tandem mass spectrometry fragmentation scan
nLC-MS/MS	Nano-Liquid Chromatography Tandem Mass Spectrometry
NPF	Necropsy palmar fascia
PA	Proteomic Analysis
PC1/PC2	Principal Component 1/2
PCA	Principal Component Analysis
PPI	Protein–Protein Interaction
PRELP	Prolargin
RC	Relapsed Clubfoot
RCT	Relapsed Clubfoot Tissue
STRING	Search Tool for the Retrieval of Interacting Genes and Proteins
TGFBI	TGF-β–inducible protein
TGF-β	Transforming Growth Factor Beta
THBS4	Thrombospondin-4
TNC	Tenascin-C
α-SMA	Alpha Smooth Muscle Actin

## Appendix A

### Appendix A.1

**Table A1:** Detailed characteristics of 12 patients with Clubfoot relapse after unsuccessful casting therapy who underwent surgical treatment.

Patient	Age (Y)	Gender	Diagnosis	Classification	Laboratory Method
1	5	M	RCT, RCT control	III	HPLC + PA
2	4	M	RCT, RCT control	III	HPLC + PA
3	6	M	RCT, RCT control	IV	HPLC + PA
4	4	M	RCT, RCT control	III	HPLC + PA
5	4	M	RCT, RCT control	III	HPLC + PA
6	7	F	RCT, RCT control	III	HPLC + PA
7	4	M	RCT, RCT control	III	HPLC
8	6	M	RCT, RCT control	IV	HPLC
9	5	M	RCT, RCT control	III	HPLC
10	3	F	RCT, RCT control	III	HPLC
11	7	M	RCT, RCT control	IV	HPLC
12	8	F	RCT, RCT control	IV	HPLC
Total 12	Mean 5.25 years (SD = 1.54)	3xF, 9xM	Relapsed Clubfoot tissue (RCT), Relapsed Clubfoot tissue control (RCT control)	Dimeglio clubfoot classification	High-performance liquid chromatography (HPLC), Proteomic analysis (PA)

### Appendix A.2

**Table A2:** Detailed characteristics of 19 patients with Dupuytren’s disease (sample donors) who underwent surgical treatment, and 10 necropsy-derived control donors providing samples of physiological necropsy palmar fascia.

Patient	Age (Y)	Gender	Diagnosis	Hand Side	Laboratory Method
1	65	F	DDN (Woodruff G3)	R	HPLC + PA + IHC
2	55	F	DDN (Woodruff G4)	L	HPLC + PA + IHC
3	59	M	DDN (Woodruff G4)	L	HPLC + PA + IHC
4	61	M	DDN (Woodruff G3)	R	HPLC + PA + IHC
5	62	F	DDN (Woodruff G4)	R	HPLC + PA + IHC
6	74	M	DDN (Woodruff G4)	L	HPLC + PA + IHC
7	45	F	DDN (Woodruff G4)	L	HPLC + IHC
8	50	M	DDN (Woodruff G3)	R	HPLC + IHC
9	48	M	DDN (Woodruff G3)	L	HPLC + IHC
10	60	M	DDN (Woodruff G4)	R	HPLC + IHC
11	63	M	DDN (Woodruff G4)	R	HPLC
12	75	M	DDN (Woodruff G3)	L	HPLC
13	67	F	DDN (Woodruff G3)	L	HPLC
14	58	M	DDN (Woodruff G4)	L	HPLC
15	70	M	DDN (Woodruff G4)	R	HPLC
16	64	M	DDN (Woodruff G3)	R	HPLC
17	58	F	DDN (Woodruff G4)	L	HPLC
18	61	M	DDN (Woodruff G4)	R	HPLC
19	49	F	DDN (Woodruff G3)	R	HPLC
Total 19	Mean 60.21 years (SD = 8.33)	7xF, 13xM	Dupuytren disease nodules (DDN)	10xR, 9xL	High-performance liquid chromatography (HPLC), Proteomic analysis (PA), Immunohistochemistry (IHC)
1	88	F	NPF	L	HPLC + PA + IHC
2	81	F	NPF	R	HPLC + PA + IHC
3	82	M	NPF	R	HPLC + PA + IHC
4	89	M	NPF	L	HPLC + PA + IHC
5	81	M	NPF	R	HPLC + PA + IHC
6	79	M	NPF	L	HPLC + PA + IHC
7	80	F	NPF	L	HPLC + IHC
8	85	F	NPF	L	HPLC + IHC
9	86	M	NPF	R	HPLC + IHC
10	78	F	NPF	L	HPLC + IHC
Total 10	Mean 75.7 years (SD = 3.84)	5xF, 5xM	Necropsy Palmar fascia (NPF)	4xR, 6xL	High-performance liquid chromatography (HPLC), Proteomic analysis (PA), Immunohistochemistry (IHC)
