# Supplementary material for: Shared Extracellular Matrix Remodeling and Proteomic Signature in Dupuytren’s Disease and Relapsed Clubfoot Tissue
Source: Cells. 2026 May 26;15(11):977. doi: 10.3390/cells15110977 (PMC13256552; doi:10.3390/cells15110977)
Supplement: Supplementary file 1 [file cells-15-00977-s001.zip › cells-4303592-supplementary.pdf]

Table S1

log_2FC (DD vs NPF)	p value	Protein name	external_gene_name
1,89437	1,172E-05	Lumican	LUM
2,61766	2,718E-05	Fibromodulin *	FMOD
2,05584	3,194E-05	Osteoglycin	OGN
2,52142	0,00030	Biglycan *	BGN
2,46740	0,00035	Collagen alpha-1(VI) chain *	COL6A1
1,91084	0,00044	Decorin	DCN
1,08048	0,00056	Collagen alpha-1(V) chain	COL5A1
2,56166	0,00060	Asporin *	ASPN
2,19796	0,00068	Collagen alpha-2(VI) chain *	COL6A2
2,65767	0,00071	Thrombospondin-4 *	THBS4
1,75781	0,00079	Collagen alpha-3(VI) chain *	COL6A3
4,75901	0,00148	Periostin	POSTN
2,71972	0,00155	Collagen alpha-1(XII) chain *	COL12A1
4,29168	0,00172	Hemoglobin subunit beta	HBB
1,59327	0,00273	Prolargin *	PRELP
3,64683	0,00504	Hemoglobin subunit alpha	HBA2
3,64683	0,00504	Hemoglobin subunit alpha	HBA1
1,99842	0,00674	Transforming growth factor-beta-induced protein *	TGFBI
1,50650	0,00757	Dermatopontin	DPT
2,08872	0,01594	Lysyl oxidase	LOX
1,84358	0,01469	Histone H2A type 1-H	H2AC14
2,72240	0,03149	Calpain-2 catalytic subunit	CAPN2
1,89068	0,02385	Dihydropyrimidinase-related protein 2	DPYSL2
1,10151	0,02024	Histone H1.0	H1-0
2,89512	0,02028	Tenascin-C *	TNC
1,00052	0,02300	Cadherin-13	CDH13
1,49779	0,02116	Annexin A2	ANXA2

2,34723	0,03309	Laminin subunit gamma-1	LAMC1
2,20750	0,02467	14-3-3 protein epsilon	YWHAE
2,24134	0,03930	Hemoglobin subunit delta	HBD
1,89597	0,02879	Vimentin	VIM
1,65922	0,02987	Phosphoglycerate kinase 1	PGK1
2,28135	0,03016	Cartilage intermediate layer protein 2 *	CILP2
1,27121	0,03085	Collagen alpha-1(IV) chain	COL4A1
2,80266	0,04345	Collagen alpha-1(VII) chain	COL7A1
2,47611	0,04756	14-3-3 protein theta	YWHAQ
1,22155	0,04969	Collagen alpha-1(XVIII) chain	COL18A1
-1,12496	0,02508	Collagen alpha-2(XI) chain	COL11A2
-1,41014	0,01447	Alpha-1-antitrypsin	SERPINA1
-1,03363	0,01689	Collagen alpha-3(IV) chain	COL4A3
-1,45481	0,02166	Lipopolysaccharide-binding protein	LBP
-1,95746	0,00779	Alpha-1-antichymotrypsin	SERPINA3
-2,69364	0,00131	Leucine-rich alpha-2-glycoprotein	LRG1

Table S1: The list of proteins with significantly changed concentration DD vs NPF. Positive difference values of log2 fold change (log_2FC) indicate significantly upregulated proteins in DD samples, and negative values of log_2FC indicate significantly downregulated proteins in DD samples. An asterisk indicates a significantly increased protein concentration in both DD and RCT (vs their controls).

Table S2

**log_2FC (RCT vs RCT control)**	**p value**	**Protein Name**	**external_gene_name**
4,05159	0,00667	Fibromodulin *	FMOD
2,99635	0,01435	Cartilage intermediate layer protein 2 *	CILP2
2,79760	0,00187	ABI family member 3-binding protein	ABI3BP
2,56897	0,01030	Collagen alpha-1(XII) chain *	COL12A1
2,55436	0,04907	Prolargin *	PRELP

2,49156	0,00506	Biglycan *	BGN
2,48794	0,00561	Cartilage oligomeric matrix protein	COMP
2,48491	0,01848	Versican core protein	VCAN
2,25198	0,03045	Cartilage intermediate layer protein 1	CILP
1,99505	0,04035	Tenascin-C *	TNC
1,93850	0,02284	Transforming growth factor-beta-induced protein *	TGFBI
1,85894	0,01074	EMILIN-3	EMILIN3
1,82294	0,01945	Collagen alpha-3(VI) chain *	COL6A3
1,75106	0,03155	Asporin *	ASPN
1,69113	0,02206	Collagen alpha-1(VI) chain *	COL6A1
1,53763	0,04467	Complement C3 *(UniProt ID P09525)*	P09525
1,47065	0,01996	Thrombospondin-4 *	THBS4
1,45876	0,01871	Collagen alpha-2(VI) chain *	COL6A2
1,32889	0,01040	Alpha-1-microglobulin/bikunin precursor	AMBP
1,26579	0,03617	Collagen alpha-1(XIV) chain	COL14A1
1,17809	0,02007	Thrombospondin-3	THBS3
-1,19040	0,01093	EMILIN-1	EMILIN1
-1,21815	0,04114	Stress-70 protein, mitochondrial (Mortalin)	HSPA9
-1,32830	0,01771	Collagen alpha-1(XVIII) chain	COL18A1
-1,41703	0,00590	Acetyl-CoA acetyltransferase, mitochondrial	ACAT1
-1,47754	0,02267	Malate dehydrogenase, mitochondrial	MDH2
-1,49944	0,02211	AP-2 complex subunit alpha-1	AP2A1
-1,51470	0,02641	Collagen alpha-3(V) chain	COL5A3
-1,51775	0,02530	Complement C1q tumor necrosis factor-related protein 5	C1QTNF5
-1,71406	0,01198	10 kDa heat shock protein, mitochondrial	HSPE1
-2,36539	0,00738	Cathepsin B	CTSB

Table S2: The list of proteins with significantly changed concentration RCT vs RCT control. Positive difference values of log_2FC indicate significantly upregulated proteins in RCT samples, and negative values of log_2FC indicate significantly downregulated proteins in RCT control samples. An asterisk indicates a significantly increased protein concentration in both DD and RCT (vs their controls).
